# Clima de aprendizaje y enganche al trabajo del residente clínico: relación con la autodeterminación humana

**DOI:** 10.7705/biomedica.6158

**Published:** 2022-03-01

**Authors:** Jorge Alberto Restrepo, Luis Carlos Domínguez, Marcelo García-Diéguez

**Affiliations:** 1 Departamento de Educación Médica, Universidad de La Sabana, Chía, Colombia Universidad de la Sabana Departamento de Educación Médica Universidad de La Sabana Chía Colombia; 2 Departamento de Educación Médica, Universidad Nacional del Sur, Bahía Blanca, Argentina Universidad Nacional del Sur Departamento de Educación Médica Universidad Nacional del Sur Bahía Blanca Argentina

**Keywords:** aprendizaje, autonomía personal, organización y administración, Learning, personal autonomy, organization and administration

## Abstract

**Introducción.:**

El clima de aprendizaje es un factor que se asocia con el compromiso hacia las actividades laborales del médico residente y el mejoramiento del bienestar estudiantil en el sitio de trabajo por medio de su autodeterminación durante la rotación clínica.

**Objetivo.:**

Determinar la relación del clima de aprendizaje medido con la escala D-RECT 35, y la autodeterminación del médico residente y su compromiso con el trabajo mediante la escala UWES 17.

**Material y métodos.:**

Se hizo un estudio correlacional de corte transversal con médicos residentes de especialidades médico-quirúrgicas que hacían la rotación clínica en el sitio de práctica y completaron los cuestionarios de medición.

**Resultados.:**

Se evaluaron 188 médicos residentes de especialidades clínicas; la mediana de la escala de clima de aprendizaje fue de 3,9/5,0; la de la escala de autodeterminación fue de 4,86/7,0, y la de la escala de compromiso laboral fue de 5,0/6,0. El clima de aprendizaje se consideró como adecuado y se encontró una relación positiva con la autodeterminación y el compromiso del médico residente con sus actividades; dichas correlaciones tuvieron significación estadística.

**Conclusiones.:**

El clima de aprendizaje adecuado se relaciona positivamente con la capacidad de comprometerse con las actividades laborales y con la autodeterminación del médico residente en entrenamiento; asimismo, favorecen el trabajo colaborativo y el acceso a la supervisión, y generan mayor autonomía, entusiasmo y dedicación a las actividades asignadas, lo cual puede incentivar mejoras en los programas educativos de los departamentos clínicos y reflejarse en una atención más segura a los pacientes.

## Abstract (en)

**Introduction:**

The learning climate is a factor associated with the clinical resident’s engagement in work activities and the improvement of students’ well-being in the workplace through their self-determination during clinical rotation.

**Objective:**

To determine the relationship between the learning climate measured with the D-RECT 35 scale and residents’ self-determination and commitment to work using the UWES 17 scale.

**Materials and methods:**

We conducted a cross-sectional correlational study with residents of surgical medical specialties in clinical rotation at the practice site and who completed the measurement questionnaires.

**Results:**

We evaluated 188 residents of clinical specialties. The median of the results in the learning climate scale was 3.9/5.0; in the self-determination scale, 4.86/7.0, and in the job engagement scale, 5.0/6.0. The learning climate was considered adequate and a positive relationship was found with self-determination and the residents’ attachment to their activities; these correlations were statistically significant.

**Conclusions:**

Adequate learning climates are positively related to the ability to engage in work activities and the self-determination of clinical residents as they favor collaborative work and access to supervision generating greater autonomy and more enthusiasm and dedication to assigned activities. This can drive improvements in educational programs in clinical departments and translate into safer patient care.

La relación del clima de aprendizaje con los factores asociados al bienestar estudiantil ha sido motivo de investigación detallada en el campo de la educación médica; se sabe que los ambientes de aprendizaje favorables se relacionan con un mejor cumplimiento de las actividades académicas y con un mayor compromiso con el trabajo estudiantil ^1^. Los estudiantes de posgrado de las especialidades médico-quirúrgicas desarrollan su residencia clínica en hospitales universitarios en donde asumen un papel laboral bajo la perspectiva de “aprender haciendo” en el sitio de práctica ^2^ y llevan a cabo actividades asistenciales propias de un trabajador, pero asociadas con el cumplimiento de las actividades de enseñanza-aprendizaje ^3^. Según lo establecido por Silkens, *et al*.,el clima de aprendizaje se define como “las percepciones compartidas de los residentes sobre aspectos formales e informales de la educación, incluidas las percepciones de la atmósfera en general, así como las políticas, prácticas y procedimientos dentro del hospital docente” ^4^, y puede ejercer un efecto sobre el compromiso con el trabajo, el bienestar estudiantil y las necesidades psicológicas básicas del médico residente, como la autonomía, la capacidad de relacionarse y la percepción de competencia clínica ^5^.

El compromiso con el trabajo ha sido definido por Bakker, *et al.*, como “la aplicación de las fortalezas del recurso humano y las capacidades psicológicas orientadas positivamente para mejorar el rendimiento del individuo en el sitio de trabajo” ^6^. Dicho compromiso puede explicarse a partir de la teoría de demandas y recursos en el trabajo, modelo conocido como JD-R, que clasifica en esas dos categorías todos los aspectos físicos, psicológicos y organizativos del trabajo que requieren esfuerzo físico o mental por parte del individuo, que lo someten a gran presión y lo obligan a consumir energía física o mental durante las actividades, así como los recursos laborales definidos como todos los aspectos físicos, psicológicos, sociales u organizativos que ayudan al individuo a obtener las metas laborales propuestas, estimulan su crecimiento personal, fomentan su propio aprendizaje y reducen las exigencias y el consumo de energía asociados con el trabajo ^7^. Entre los recursos laborales se incluye el logro de la autonomía, la realimentación sobre el desempeño, y el acceso a la supervisión y capacitación, entre otros.

La teoría de la autodeterminación alude a la motivación humana y fue expuesta por Ryan, *et al.*, en el año 2000 ^8^. En ella se plantea que todo individuo requiere satisfacer sus necesidades psicológicas básicas accediendo a una competencia que potencie su capacidad para relacionarse y obtener autonomía. En ese contexto diversos factores se relacionan positiva o negativamente con la satisfacción psicológica, lo que influye en la motivación y en el compromiso con el trabajo para realizar las tareas y alcanzar las metas cotidianas ^9^.

Un clima de aprendizaje óptimo puede contribuir a mejorar el compromiso con el trabajo y la autodeterminación del residente durante sus rotaciones clínicas. La medición del clima de aprendizaje ha sido motivo de múltiples estudios y son varios los instrumentos validados para tal fin. El más empleado recientemente es el *Dutch Residency Educational Climate Test*, D-RECT 35, que fue traducido al español por Domínguez, *et al.*^10^. Sus nueve dominios y 35 preguntas tienen una estructura factorial muy confiable, por lo que se eligió como instrumento de medición en este estudio. Por otra parte, Nordquist ha planteado la necesidad de dilucidar la relación entre el clima de aprendizaje en el ámbito clínico y los esfuerzos para garantizar una atención segura a los pacientes ^11^.

Asimismo, el concepto de comunidades de práctica clínica descrito por Cruess, *et al.,* postula un modelo de enseñanza durante la rotación clínica que armonice la forma en que se moldea el clima de aprendizaje, lo cual es crucial para determinar su relación con el compromiso hacia las actividades académicas y asistenciales del médico residente ^12^. González, *et al.*, por su parte, exponen las teorías educativas que dan el soporte teórico al concepto de comunidades de práctica ^13^.

En este contexto, el objetivo del estudio fue establecer la relación entre el clima de aprendizaje, y el compromiso con el trabajo y la autodeterminación del médico residente durante sus rotaciones en el sitio de trabajo, con énfasis en los estudiantes de primer y segundo año de residencia.

## Materiales y métodos

### Diseño del estudio, modelo de investigación y participantes

**Figura 1:**
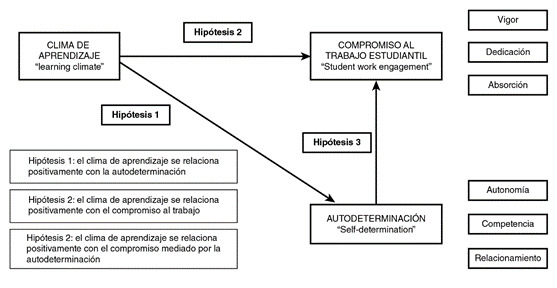
Modelo de investigación - hipótesis por probar

Se hizo un estudio correlacional explicativo, con una muestra conformada por estudiantes de los programas de postgrado de las especialidades médico-quirúrgicas de la Universidad de La Sabana en Chía, Colombia, que cursaban su rotación clínica en el sitio de práctica; la selección muestral se estableció por conveniencia a partir de los residentes de los 16 programas de especialidades médico-quirúrgicas de la Universidad. Se diseñó un modelo para probar tres hipótesis y determinar la relación positiva del clima de aprendizaje con la autodeterminación y el compromiso hacia las actividades laborales (figura 1).

### Instrumentos de recolección de información

Los instrumentos de recolección de información fueron respondidos en sesión presencial por los propios participantes en formato impreso y en presencia del investigador principal.

### Escala de medición del clima de aprendizaje D-RECT 35

Esta escala desarrollada por Boor, *et al.*, en los Países Bajos ^14^ para medir el clima de aprendizaje en estudiantes de posgrado fue validada en español por Domínguez, *et al*., en la Universidad de La Sabana ^10^.

Como ya se mencionó, consta de 35 preguntas agrupadas en nueve dominios: ambiente educativo, trabajo en equipo, rol del tutor especializado, entrenamiento y evaluación, educación formal, colaboración entre pares residentes, trabajo adaptado a la competencia de los residentes, accesibilidad de los supervisores y entrega de turno.

Como parte de la revisión de la estructura interna de la escala, se efectuó un análisis factorial para verificar la validez de constructo, la consistencia y varianza de los ítems, y su correspondencia con la conformación ‘multidominio.’

Mediante el programa SPSS 26 se calculó la adecuación de la muestra con el índice de Káiser-Meyer-Olkin (satisfactorio si es mayor de 0,80), el cual fue de 0,903; la matriz de correlaciones mostró que los ítems estaban fuertemente interrelacionados y conformaban los nueve dominios descritos.

### Escala de necesidades psicológicas básicas: autodeterminación

Esta escala fue desarrollada por Ryan, *et al.*^15^. El cuestionario está compuesto por 21 ítems que miden el grado de satisfacción de las personas en cuanto a las necesidades psicológicas básicas y fue validada en español por Delgado en la Universidad de Nuevo León, Monterrey, México ^16^; está conformada por tres dominios que miden la autonomía, la competencia y las relaciones en el trabajo.

Se revisó la estructura interna de la escala y se hizo un análisis factorial para verificar la validez de constructo, la coherencia y la varianza de los ítems, así como su correspondencia con la conformación multidominio. Mediante el programa SPSS 26, se calculó la adecuación de la muestra con el índice de Káiser-Meyer-Olkin, el cual fue de 0,707 y se consideró satisfactorio cuando era mayor de 0,80.

### Escala de compromiso con las actividades laborales (Utrecht Engagement Scale, UWES 17)

La versión original de esta escala fue desarrollada en el 2000 en la Universidad de Utrecht por Schaufeli, *et al.*^17^. Los autores emplearon el término *“work engagement*” y lo propusieron como un nuevo paradigma contrario al concepto de desgaste o *burnout*. La escala fue validada en español por un grupo de la Universidad de Concepción en Chile ^18^ y consta de 17 preguntas divididas en tres dominios: vigor, dedicación y absorción.

Se revisó la estructura interna de la escala y se efectuó un análisis factorial para verificar la validez de constructo, la coherencia y la varianza de los ítems, así como su correspondencia con la conformación multidominio. Mediante el programa SPSS 26, se calculó la adecuación de la muestra con el índice de Káiser-Meyer-Olkin, el cual fue de 0,756 y se consideró satisfactorio cuando era mayor de 0,80

### Consideraciones éticas

Este estudio se consideró de riesgo menor que el mínimo. Se solicitó la firma voluntaria del consentimiento informado a los participantes y se garantizó el manejo de los datos de forma completamente anónima. El proyecto fue sometido a revisión de pares en la Subcomisión de Educación Médica de la Facultad de Medicina de la Universidad de La Sabana.

### Análisis estadístico

Se hizo un análisis descriptivo de los datos; las variables cualitativas se presentan como frecuencias absolutas y relativas. Dada la no normalidad de los datos evaluada mediante la prueba de Shapiro-Wilk, las variables cuantitativas se presentan como medianas y rangos intercuartílicos. Se utilizó el coeficiente de correlación de Spearman para determinar la correlación simple entre los puntajes totales de las escalas.

Se utilizó un modelo de ecuaciones estructurales estandarizado para determinar la asociación directa y la indirecta, entre el clima de aprendizaje y el compromiso con el trabajo y la autodeterminación. Se analizaron por separado los puntajes globales de cada escala y sus dominios. La bondad de ajuste del modelo se evaluó por medio de los siguientes indicadores: el estadístico de ji al cuadrado, el índice de ajuste comparativo (IAC), la raíz del *error* cuadrático medio (RECM), la raíz cuadrada media residual estandarizada (*Standardized Root Mean Square Residual*, SRMR) y el valor cuadrático medio ponderado (*Weighted Root Mean Square Residual*, WRMR). En todos los casos, se consideró un valor de p inferior a 0,05 como estadísticamente significativo.

## Resultados

### Variables descriptivas y demográficas

En total, 188 residentes de 16 programas de postgrado en especialidades médico-quirúrgicas de la Universidad de La Sabana completaron los cuestionarios; la edad promedio de los participantes fue de 28 años, el 57 % correspondió a mujeres y la mayoría eran residentes de primer año, con el 67 %, y de segundo año, con el 19 % (cuadro 1). No se encontraron diferencias en cuanto al género.

**Cuadro 1:** Características demográficas de los participantes

Variable	Mediana (RIC)
Edad	28 (26-30)
Sexo*	
Femenino	105 (56,8)
Masculino	83 (43,2)
	
Año de especialidad*	
1	121 (67,2)
2	34 (18,9)
3	17 (9,5)
4	8 (4,4)

*n(%)

### Puntajes de escalas y coeficientes de correlación

Las medianas del puntaje de la escala D-RECT fueron de 3,9/5,0 (RIC=3,6-4,3), y las de la escala UWES, de 5,0/6,0, (RIC=4,3-5,5), puntajes estos adecuados según lo establecido por Domínguez, *et al.*^10^. El puntaje de la escala de necesidades psicológicas básicas fue de 4,8/7,0 (RIC=4,6 - 5,1); los coeficientes de correlación indicaron que el clima de aprendizaje se asoció positivamente con el compromiso hacia las actividades diarias y la autodeterminación; todas las correlaciones fueron estadísticamente significativas, aunque el poder de la asociación no fue fuerte (cuadro 2).

**Cuadro 2:** Puntajes y coeficientes de correlación de las escalas de medición

Escalas	Medianas
Escala D-RECT 35 (clima)	3,9/5,0 (3,6-4,3)
Escala UWES 17 (enganche)	5,0/6,0 (4,3-5,5)
Escala de necesidades psicológicas (autodeterminación)	4,8/7,0 (4,6-5,1)
**Coeficientes de correlación**	
Variable	Coeficiente
Clima - Enganche	0,2195
Clima - Autodeterminación	0,4385

El puntaje más alto en los dominios de la escala de clima de aprendizaje se registró en el trabajo colaborativo entre residentes, y el más bajo correspondió a la orientación y evaluación del supervisor. El dominio con puntaje más alto en la escala de necesidades psicológicas básicas fue el de autonomía, con 5,18, y el más bajo fue el de competencia, con 4,77; el dominio de la escala UWES 17 con el puntaje más alto fue el de dedicación, con 5,12/6,0, y el más bajo fue el de la absorción, con 4,59 (cuadro 3).

**Cuadro 3:** Puntajes de los dominios en las escalas de medición (medianas)

Dominios de la escala D-RECT 35 (clima de aprendizaje)	
1. Entorno educativo	3,78 (3,59-3,95)
2. Trabajo en equipo	3,90 (3,74-4,07)
3. Coordinación del equipo	3,80 (3,61-3,98)
4. Orientación y evaluación	3,75 (3,54-3,96)
5. Educación formal	3,95 (3,75-4,14)
6. Trabajo colaborativo entre residentes	4,20 (4,04-4,37)
7. Adaptación a las competencias	4,08 (3,90-4,25)
8. Acceso a supervisión	4,16 (3,97-4,35)
9. Entrega de turno	3,80 (3,55-4,05)
**Dominios de la escala de necesidades psicológicas (autodeterminación)**	
Competencia	4,77 (4,55-4,99)
Autonomía	5,18 (4,99-5,37)
Relaciones	4,90 (4,72-5,07)
**Dominios de la escala UWES 17 (compromiso)**	
1. Vigor	4,67 (4,62-4,72)
2. Dedicación	5,12 (5,11-5,13)
3. Absorción	4,59 (4,53-4,65)

El modelo de ecuaciones estructurales mostró que había una relación directa entre el clima laboral y el compromiso con el trabajo (coeficiente estandarizado: 0,16; p=0,039), y entre el clima de aprendizaje y la autodeterminación (coeficiente estandarizado: 0,47; p<0,01). Asimismo, se evidenció que la relación entre el clima de aprendizaje y el compromiso con las actividades del residente mediado por la autodeterminación también fue estadísticamente significativa (coeficiente estandarizado: 0,47 * 0,28 = 0,1269; p<0,01), y que hubo un efecto parcial entre el clima de aprendizaje y el compromiso con el trabajo mediado por la autodeterminación comparable con el efecto directo entre el clima y el compromiso con el trabajo (0,13 *Vs*. 0,16). Los indicadores evidenciaron que el modelo presentó un buen ajuste (CFI=1,0; RMSEA=0,0; SRMR=0,0), y todos ellos fueron estadísticamente significativos (p<0,01).

## Discusión

El estudio aportó evidencia de una relación positiva entre el clima de aprendizaje y las percepciones de autodeterminación de los residentes de especialidades médico-quirúrgicas, lo que confirma la hipótesis 1 del modelo de investigación planteado. Asimismo, se halló evidencia de que el clima de aprendizaje se relacionó positivamente con la capacidad de comprometerse con las actividades diarias, confirmándose así la hipótesis 2. El estudio también confirmó la hipótesis 3, al encontrar una relación positiva entre el clima de aprendizaje y el compromiso con el trabajo del médico residente mediado por la autodeterminación. La bondad de ajuste del modelo fue adecuada y se corroboró la validez de las correlaciones encontradas.

En su estudio, Lases, *et al*., observó una fuerte correlación entre el clima de aprendizaje medido con la escala D-RECT y el bienestar global del residente como reflejo del compromiso con las actividades diarias, predominantemente en los dominios de entorno educativo, trabajo en equipo, papel del coordinador y colaboración entre pares, lo que coincide con nuestros hallazgos ^19^. Ello permite plantear que el verdadero bienestar del residente no depende solamente de tener horas de trabajo balanceadas, o descanso adecuado después de los turnos nocturnos, o una remuneración, alimentación o esparcimiento justos, que, aunque importantes, no son los que influyen finalmente en el desarrollo de una verdadera competencia clínica del médico residente y de su identidad profesional, o en la generación de cambios en su práctica profesional. Una mayor confianza mejora las relaciones y la percepción de competencia de las personas en el desarrollo de cualquier actividad que se les asigne dentro de un equipo de trabajo a partir del estudio de la motivación humana desde el punto de vista global, contextual y situacional ^20^.

Los modelos de correlación aplicados mostraron que, cuanto mejor era el clima de aprendizaje, mayor la autodeterminación de los residentes y el compromiso con sus actividades laborales; sin embargo, el nivel de correlación fue bajo en las puntuaciones del dominio de vigor de la escala de medición del compromiso, lo que podría relacionarse con la sensación de cansancio extremo o la poca motivación ante las tareas asignadas, pues la pesada carga laboral durante la rotación genera un gran consumo de energía. En las puntuaciones de la escala de medición del clima de aprendizaje no hubo una fuerte asociación con el dominio de supervisión, lo que denota que los profesores a cargo tal vez no toman la iniciativa para evaluar el desempeño de los residentes y no hacen mayor seguimiento a sus actividades, lo que, en consecuencia, los desmotiva.

Levesque-Bristol observaron que la motivación intrínseca era el factor que promovía el aumento de la autodeterminación a partir de los tipos de clima que se generaban alrededor del aprendizaje y de la supervisión y orientación dada por el profesor, pues al tener una mejor disposición con el trabajo cooperativo, los residentes se encargaban de cumplir con todas las asignaciones diarias y lograban satisfacer sus necesidades psicológicas básicas ^21^. Estas observaciones conducen a plantear que se requiere mayor dedicación por parte de los profesores clínicos para generar mejores elementos educativos que modulen estas características, con el fin de apuntalar la sensación de competencia del residente.

Desde la perspectiva del modelo de demandas y recursos en el trabajo (JD-R), que los residentes cuenten con acceso a supervisión, realimentación al desempeño, acompañamiento, andamiaje y articulación de conocimientos, son los factores que sirven para “contrarrestar” las demandas académicas y laborales asignadas, como son la alta carga laboral y administrativa durante los turnos, tener que realizar procedimientos complejos, atención a múltiples pacientes, tener que lidiar con situaciones de fracaso en desenlaces clínicos, abordaje a las familias de los pacientes y relaciones con los equipos de trabajo. Cuando se ofrecen pocos recursos a los residentes y se les hacen muchas exigencias o demandas, estos pueden acusar falta de energía, lo que se refleja en poco vigor durante el trabajo; por el contrario, cuando esos recursos son bien administrados, así se tengan muchas exigencias, el residente desarrolla mucha dedicación y absorción por el trabajo que lo llevan a lograr metas, aprovechar oportunidades de aprendizaje y cuidar mejor la salud de los pacientes ^22^.

Se debe aclarar que el concepto de compromiso es el contrario al de desgaste o *burnout* y que algunas mediciones relacionan el clima de aprendizaje con síndromes de desgaste. Llera, *et al*., evaluaron la relación del clima educacional y el síndrome de desgaste en estudiantes de internado y encontró que, cuando el clima de aprendizaje era deficiente, había mayores niveles de agotamiento, en tanto que con puntajes altos había mayor autonomía ^23^. En el presente estudio, se hizo una aproximación a la problemática desde el lado contrario al desgaste, es decir, el compromiso, y también se encontró una clara correlación entre los puntajes altos en las escalas de medición del clima de aprendizaje y los relativos al compromiso frente al trabajo.

El modelo de comunidades de práctica clínica puede favorecer el compromiso laboral. En este sentido, Cruess, *et al*., señalan que el residente puede ingresar a las comunidades de práctica clínica participando desde “la periferia”, sin comprometerse mucho con la actividad laboral, y a medida que adquiere habilidades y asume más responsabilidades se mueve hacia “el centro” hasta llegar a obtener una “membresía” que legitima su participación dentro de la comunidad, lo cual fomenta la confianza paulatinamente, pues la adquisición de conocimientos y habilidades es gradual. En ambientes de aprendizaje óptimos, se incentiva el compromiso con el trabajo asignado hasta obtener una participación central que fortalecerá su vigor y su dedicación al trabajo durante la rotación ^12^.

El presente estudio adolece de un equilibrio en la distribución del año de residencia de los participantes, pues hubo un claro predominio de residentes de primer año, lo cual implica que la mayoría eran novatos y todavía no habían afianzado su participación en la rotación clínica y apenas empezaban a construir su identidad como especialistas. Ello abre la posibilidad de estudios futuros para observar cómo evoluciona en los médicos residentes su compromiso con el trabajo, y la forma en que van adquiriendo autonomía y confianza a medida que se adaptan a la rotación clínica, lo que permitiría mejorar los modelos de enseñanza en los sitios de práctica y lograr que se conformen verdaderas comunidades de práctica clínica.

Otra debilidad del estudio radicó en se hicieron mediciones en múltiples especialidades médico-quirúrgicas que implican diferentes tipos de clima de aprendizaje. Silkens, *et al*., demostraron una asociación fuerte entre un buen clima de aprendizaje y la organización de la enseñanza en 17 departamentos clínicos en los Países Bajos ^24^ y encontró que predominaron cuatro tipos de clima que definió como deficientes, adecuados, buenos y excelentes.

Se ha planteado, asimismo, la posibilidad de desarrollar nuevas mediciones del clima de aprendizaje que sirvan para optimizar la rotación clínica, las condiciones y estándares de calidad de la enseñanza del propio departamento médico-quirúrgico de que se trate, y una atención segura al paciente ^25^. Por su parte, Smirnova, *et al*., estudiaron la relación del clima de aprendizaje y los eventos adversos o complicaciones en un departamento de obstetricia, y observaron que el clima de aprendizaje se asociaba con mayores probabilidades de resultados perinatales adversos, pero no maternos, aunque advirtieron que se requerían nuevos estudios para determinar los mecanismos que subyacían a esas asociaciones ^26^.

Los hallazgos del presente estudio abren el camino a nuevas incursiones en el tema, ya que las evidencias recolectadas sobre la relación del clima de aprendizaje con el compromiso hacia el trabajo mediado por la autodeterminación, posibilitan evaluar globalmente el clima de aprendizaje en los diferentes departamentos clínicos de las instituciones educativas u hospitalarias; asimismo, estos hallazgos pueden abrir nuevos caminos para medir la relación entre el clima de aprendizaje y la seguridad en la atención de los pacientes.
